# Converging Issues in Veterinary and Public Health

**DOI:** 10.3201/eid0904.030037

**Published:** 2003-04

**Authors:** Lonnie King, Rima Khabbaz

**Affiliations:** * Michigan State University College of Veterinary Medicine, East Lansing, Michigan, USA; † Centers for Disease Control and Prevention, Atlanta, Georgia, USA

More than 20 key officials from the American Veterinary Medical Association and the Association of American Veterinary Medical Colleges met with staff from the Centers for Disease Control and Prevention (CDC) December 5–6, 2002, to discuss the increasing convergence of issues confronting human and animal health. Among the officials in attendance were the deans from more than half of all U.S. veterinary schools.

The meeting goals were to increase the veterinary community’s understanding of CDC programs and the varied roles played by veterinarians throughout the agency; to provide CDC officials an opportunity to gain insight into current issues in veterinary medicine as well as the public health perspectives of veterinary leaders; and to provide a forum for discussions on ways to increase partnerships between the human and veterinary medical communities to meet critical public health needs. Presentations were made by James Hughes, Michel Bunning, Patricia Griffin, David Bell, Nina Marano, Tracee Treadwell, Thomas Ksiazek, and Peter Schantz, National Center for Infectious Diseases; Marguerite Pappaioanou, Office of Global Health; Hugh Mainzer, National Immunization Program; Douglas Hamilton, Epidemiology Program Office; and Andrew Dannenberg, National Center for Environmental Health. Many of these speakers are CDC veterinarians, who described their paths from veterinary training to public health.

The daily interactions of humans, animals, and the environment have a dramatic impact on public health. Current and evolving health threats include infections transmitted through animals, insects, food, and water, as well as illnesses resulting from environmental toxins, the misuse of antibiotics, and bioterrorism. Factors affecting these threats include the international movement of people, animals, and animal products; globalization and management of the complex food and fiber system; climate and other environmental changes, including those affecting wildlife populations and their interactions; and national and global security. Effectively meeting these challenges requires strong links between human and animal health clinicians, researchers, laboratorians, and public health officials.

Specific topics presented included West Nile virus and other vectorborne diseases, emerging viral and parasitic zoonoses, food safety, antimicrobial resistance, CDC’s role in the 2001 anthrax investigations, and the agency’s bioterrorism preparedness and response program. Presentations highlighted public health issues such as the need to upgrade containment facilities and to define optimal antibiotic use for farm animals. Efforts needed to further protect the health of humans, companion animals, zoo and exotic animals, and wildlife were also discussed. These efforts include improving strategies to reduce the occurrence of intestinal parasites in pets and increasing surveillance among imported animals and products to recognize infections not previously seen in the United States.

Several presenters emphasized the importance of surveillance systems in enabling prompt recognition of disease occurrences. Examples included two food safety surveillance programs: FoodNet, a collaborative project involving nine states, the U.S. Department of Agriculture, CDC, and the Food and Drug Administration; and PulseNet, a national and international network of public health laboratories that subtype foodborne bacteria to enable rapid comparison of DNA patterns through an electronic database. Other surveillance systems discussed included the National Antimicrobial Resistance Monitoring Systems and the Laboratory Response Network (LRN). LRN is a tiered system of laboratories with varying diagnostic capabilities, ranging from confirmatory analysis to specialized identification of agents potentially used in a bioterrorist attack. The network is supported through funding designated for bioterrorism preparedness and response. Meeting participants discussed the need to increase participation of veterinary clinicians and diagnosticians in these surveillance systems, especially LRN, noting that 80% of the agents classified as “category A” (i.e., those posing a major risk to national security because they can be easily disseminated or transmitted from person to person, result in high death rates, and require special efforts to ensure preparedness) are zoonotic. Strategies discussed at the conference toward this end included adding veterinary and animal health laboratories to LRN as well as establishing a similar network among such laboratories to collect more comprehensive data on the occurrence of infections affecting veterinary and human health.

CDC veterinarians participating in the meeting described their experiences as well as the roles of other agency veterinarians. Many CDC veterinarians are epidemiologists who joined the agency as officers in the Epidemic Intelligence Service (EIS), CDC’s 2-year, hands-on comprehensive epidemiology and public health training program. Of the approximately 75 veterinarians who work at CDC, nearly half are in the National Center for Infectious Diseases, where they work in laboratory animal research as well as epidemiology. Discussions at the meeting described the critical roles played by veterinarians at the local, state, and national levels in responding to the recent West Nile virus outbreaks. Approximately 42 states currently have state public health veterinarians.

Many discussions focused on ways to increase the number of veterinarians in public health clinical and laboratory programs. Several CDC veterinarians cited classes in herd health as stimulating their interest toward public health careers. At the initial level, efforts are needed to ensure that veterinary students are aware of these career opportunities early in their education. Potential strategies include offering externships and public health rotations, such as at CDC or at local and state health departments, as part of veterinary medical school training courses and offering combined degrees in veterinary medicine and public health (i.e., DVM/MPH)—a course of study already offered by several veterinary colleges. Other innovative public health programs that could be incorporated by veterinary medical colleges include studies in food safety, environmental toxicology, healthy ecosystems, international diseases, and population medicine.

More veterinary EIS Officers are also needed. Approximately one third of veterinarians applying to EIS are accepted, essentially the same acceptance rate as for other professions. Increased numbers of veterinarian applicants would therefore translate into higher numbers of accepted veterinarians. EIS recruits at the national American Veterinary Medical Association meeting but could expand its efforts to include schools of veterinary medicine. Similarly, efforts are needed to increase the number of veterinarians and veterinary students applying for other training programs at CDC such as the Emerging Infectious Diseases Laboratory Fellowships. Through this program, bachelor’s or master’s level scientists are recruited for 1-year assignments and postdoctoral level scientists for 2-year assignments at state, local, and CDC public health laboratories. More veterinary applicants are also needed for other training programs offered by CDC, such as the elective in epidemiology for senior medical and veterinary students—a 6- to 8-week introductory course in preventive medicine, public health, and applied epidemiology.

The World Health Organization (WHO) will soon be making available on the Internet its findings from a study group on the future of veterinary public health (WHO Technical Report Series 907). The report describes the increasing emergence and reemergence of zoonotic diseases in the 1980s and 1990s and their importance for global public health (*1*).

To effectively meet these challenges, human and animal health issues must be merged into a new public health agenda. Creating and responding to such an agenda depend on strong interactions between the human and veterinary clinical, laboratory, and public health professional organizations. These interactions are essential for developing new and strengthening existing partnerships necessary for implementing effective public health programs. This meeting was a step toward this goal.
